# Clinicopathologic Characteristics and Outcomes of Simultaneous Multiple Primary Lung Cancer

**DOI:** 10.1155/2021/7722231

**Published:** 2021-12-23

**Authors:** Ying Liu, Yu-Ping Zhou, Mai Zhang, Li Li, Hu Liao, Lin Ma, Feng Lin, Yue-Yun Chen, Chun-Xi Fu, Ting-Ting Huang, You Lu, Yan Zhang

**Affiliations:** ^1^Department of Thoracic Oncology, State Key Laboratory of Biotherapy and Cancer Center, West China Hospital, Sichuan University, Chengdu, Sichuan, China; ^2^The Comprehensive Cancer Center of Drum Tower Hospital, Clinical Cancer Institute of Nanjing University, Nanjing, China; ^3^Department of Cardiology, State Key Laboratory of Complex Severe and Rare Diseases, Peking Union Medical College Hospital, Chinese Academy of Medical Science, Peking Union Medical College, Beijing, China; ^4^Department of Thoracic Surgery, West China Hospital, Sichuan University, Chengdu, Sichuan, China; ^5^Department of Biotherapy, Cancer Center, West China Hospital, West China Medical School, State Key Laboratory of Biotherapy, Sichuan University, Chengdu, Sichuan, China; ^6^Department of Pediatric Hematology and Oncology, West China Second University Hospital, Sichuan University, Chengdu, Sichuan, China; ^7^Department of Pneumology, Peking Union Medical College Hospital, Chinese Academy of Medical Science and Peking Union Medical College, Beijing, China

## Abstract

**Background:**

Simultaneous multiple primary lung cancer has been detected increasingly nowadays with the development of image technology. However, the clinicopathologic characteristics and outcomes are not clear.

**Methods:**

All consecutive patients diagnosed as simultaneous multiple primary lung cancer according to Martini–Melamed and American College of Chest Physicians criteria from June 2010 to June 2019 in our center were enrolled. The clinicopathologic characteristics and outcomes were compared between patients with the same histological type and different histological types.

**Results:**

A total of 336 patients were enrolled, consisting of 297 (88.4%) patients with the same histological type and 39 (11.6%) patients with different histological types. Compared to patients with the same histological type, patients with different histological types were more commonly males (87.2% vs. 34.0%; *p* < 0.001) with an older age (65 [62–69] vs. 59 [52–65] yrs; *p* < 0.001) at diagnosis. Also, patients with different histological types showed worse respiratory function and more advanced stage according to TNM staging. The 1-, 2-, and 3-year overall survival of overall patients was 97.7%, 96.1%, and 92.2%, and the 1-, 2-, and 3-year recurrence-free survival of overall patients was 96.8%, 92.9% and 85.7%, respectively. Importantly, patients with different histological types showed worse overall survival (*p* < 0.001) and recurrence-free survival (*p*=0.002) than patients with same histological type. The multivariable Cox proportional hazard model revealed that presence of different histological types was significant predictor for worse overall survival (adjusted hazard ratio: 10.00; 95% confidence interval: 2.92–34.48; *p* < 0.001) and recurrence-free survival (adjusted hazard ratio: 2.59; 95% confidence interval: 1.14–5.88; *p*=0.023).

**Conclusions:**

Although relatively less common in simultaneous multiple primary lung cancer, patients with different histological types showed worse clinical characteristics and outcomes.

## 1. Introduction

Multiple primary lung cancer (MPLC) refers to the occurrence of two or more primary lung cancers in one or both lungs at the same time or successively. According to the time interval of different cancer lesions, MPLC was divided into simultaneous MPLC (sMPLC) and metachronous MPLC (mMPLC). The criteria for the diagnosis of MPLC was first established by Martini and Melamed [1] and later developed by the American College of Chest Physician (ACCP) [2]. Both criteria are based on clinicopathologic and radiological features of lung nodules and have been in mainstream use due to their practicality, especially for preoperative evaluation. Notably, recent attempts of incorporating molecular and histological profiles into the diagnosis suggested their superiorities in distinguishing between MPLC and intro-pulmonary metastasis, which showed promising prospects [3–6].

With the use of high-resolution chest imaging system and lung cancer screening program, patients with MPLCs are becoming a growing population in clinical practice worldwide, especially for sMPLC [7–9]. In patients with sMPLC, the histological types of different lesions are critical and patients can be divided into two groups, including patients with the same histological type and patients with different histological types. There were some studies previously reported the proportion of patients with different histological types in sMPLC. However, due to the limited sample size and different population of reported studies, the proportion ranged from 3.8% to 61.5% in reported studies [10–13]. In addition, there was rare evidence about the differences of clinicopathologic characteristics and outcomes between patients with the same histological type and different histological types.

So, the aim of the present study was to investigate the proportion of patients with different histological types in sMPLC and confirm the differences of clinicopathologic characteristics and outcomes between patients with the same histological type and different histological types.

We present the following article in accordance with the STROBE Reporting Checklist.

## 2. Methods

### 2.1. Study Design and Participants

From June 2010 to June 2019, all consecutive patients diagnosed as sMPLC after surgery resection in our center according to Martini–Melamed [1] and American College of Chest Physicians criteria [2] were enrolled in this cohort study. All resected lesions were conducted with pathological examination to investigate the histological type. These patients were classified into two groups according to the histological types of lesions from the same patient, including sMPLC with the same histological type and sMPLC with different histological types. The demographic, clinical, pathological characteristics, and outcomes data of the included patients were collected and compared between the two groups. The ethics committee of our hospital approved this study and written informed consent was obtained from all participants.

### 2.2. Surgery Strategy

All enrolled patients underwent focal surgery resection plus systematic lymph node dissection and sampling. The surgery strategy was selected according to the size and location of lesions, the result of frozen section, the age, the pulmonary function, and basic physical condition of the patients [14]. Video-assisted thoracoscopic surgery was the main method. If the lesions were located on the ipsilateral side, they will be resected at the same time. Otherwise, staging operation was recommended. The surgery methods consisted of single lobectomy, multiple lobectomy, single sublobar resection, multiple sublobar resection, lobectomy plus sublobar resection, and total pneumonectomy. If the lesion was located in the same segment or same lobe, single sublobar resection and single lobectomy were used, respectively; when the lesions are located in different lung lobes, sublobar resections, lobectomies, and even total pneumonectomy were considered. In patients with multiple nodules with a dominant solid lesion, lobectomy of the dominant nodule along with sublobar resection of other nodules was the common procedure.

### 2.3. Clinical Assessment

All enrolled patients received a comprehensive assessment before surgery, including symptom inquiry, physical examination, laboratory test, chest radiograph, cardiopulmonary function test, chest and abdominal CT scan, brain MRI, and bone scan. Each tumor was staged separately according to the revised TNM system [15] and the most advanced disease stage of all tumors was used as final disease stage of the patient. The clinicopathologic data including age, gender, symptom, smoking history, respiratory function, laboratory test, tumor histology, tumor location, and size were investigated.

### 2.4. Molecular Analysis

Somatic mutations in epidermal growth factor receptor (EGFR) were tested with resected specimen using either amplification refractory mutation system (ARMS) including common EGFR mutations (covering 29 known mutations in exons 18–21) or the next-generation sequencing. Somatic mutations in Kirsten-rat sarcoma 2 viral oncogene homolog (KRAS) were tested with the next-generation sequencing. Immunohistochemistry was performed for detection of programmed cell death ligand 1 (PD-L1) and PD-L1 positive was defined as membranous staining present in >1% of the cells. The anaplastic lymphoma kinase (ALK) and ROS proto-oncogene 1 (ROS1) immunohistochemistry assay were conducted and a binary scoring system was used to evaluate the staining results. The presence of strong granular cytoplasmic staining in tumor cells (any percentage of positive tumor cells) was considered as positive, and the absence of strong granular cytoplasmic staining in tumor cells was considered as negative.

### 2.5. Follow-Up

Patients were routinely followed up after surgery by telephone interview or clinic visit until 30 November 2019. Chest and abdominal CT scan was performed every 3 months from 1 to 2 years after operation, and every 6 months from 3 to 5 years after operation, and every 12 months after 5 years. The brain MRI and bone scan could be added according to the changes of the patient's condition. The overall survival (OS) was estimated from the date of surgery until death of any cause or the date of last follow-up. Recurrence-free survival (RFS) was defined as the time from the date of surgery to the first event, including recurrence and metastasis, or last follow-up.

### 2.6. Statistical Analysis

Continuous variables were presented as median (interquartile range (IQR)) and compared using the Mann–Whitney *U* test. Categorical variables were summarized by number (proportion) and compared with chi-square test or Fisher exact test. The OS curves and RFS were plotted using Kaplan–Meier method and compared with the log-rank test. A multivariable Cox proportional hazard model was used to evaluate potential factors associated with OS and RFS. Variables which demonstrate significant association with the outcome in univariable analysis were candidates for further multivariable analysis. Variable selection in final parsimonious multivariable model was based on a forward-stepwise selection procedure. In all the analyses, a 2-tailed *p* value < 0.05 was considered statistically significant. All data were analyzed by using Statistic Package for Social Science (SPSS Inc., Chicago, USA) version 23.

## 3. Results

### 3.1. Enrolled Patients

**Figure 1:**
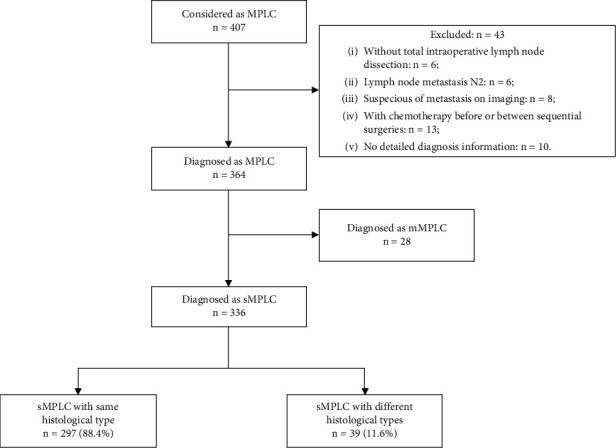
Flow chart. MPLC = multiple primary lung cancer; mMPLC = metachronous multiple primary lung cancer; sMPLC = simultaneous multiple primary lung cancer.

From June 2010 to June 2019, a total of 407 patients were considered as the diagnosis of MPLC. Forty-three patients were excluded, thus 364 patients were definitely diagnosed as MPLC. Among patients with MPLC, 28 were mMPLC. Finally, 336 sMPLC patients were enrolled. Among the enrolled patients, multiple lesions with the same histological type were recorded in 297 patients (88.4%), whereas multiple lesions with different histological types were detected in the other 39 patients (11.6%) (Figure 1).

### 3.2. Baseline Characteristics

The baseline clinical and histological characteristics are shown in Table 1 and Table 2, respectively. The median age of overall patients was 63 (56–70) years old and female was dominant (61.6%). Most patients (69.0%) were diagnosed with no symptoms and a smoking history was detected in 88 (26.2%) patients. A total of 246 (73.2%) patients had 2 lesions and only 13 (3.9%) patients had more than 4 lesions. For histological characteristics, adenocarcinoma was the most common histological type, and most were ranging from stage I A to I B according to TNM staging.

Compared with patients with the same histological type, patients with different histological types were older during diagnosis (65 [62–69] vs. 59 [52–65]; *p* < 0.001) and less commonly females (12.8% vs. 68.0%; *p* < 0.001). In addition, more patients with different histological types suffered from respiratory symptom and/or pain at diagnosis (56.4% vs. 27.6%; *p* < 0.001). Also, smoking history was more common in patients with different histological types (79.5% vs. 16.8%; *p* < 0.001). Importantly, patients with different histological types showed worse respiratory function with lower forced expiratory volume in 1 second (FEV1) and diffusing capacity for carbon monoxide (DLCO). The inflammation biomarkers were analyzed, including serum lactate dehydrogenase (LDH), neutrophile granulocyte, and derive neutrophil-to-lymphocyte radio (dNLR). Patients with different histological types showed higher dNLR and neutrophile granulocytes. Interestingly, patients with different histological types had more pleural invasion (56.4% vs. 29.0%; *p* < 0.001) and showed a more advanced stage according to TNM staging (*p* < 0.001).

### 3.3. Surgical Strategy

The surgical strategy is summarized in Supplemental Table 1. Only 74 (22.0%) patients received sequential surgeries and the median surgery interval was 7 [4–17] months. Most patients (73.5%) received thoracoscopic surgery and 18 (5.4%) patients received both thoracoscopic surgery and open heart surgery. Lobectomy plus sublobar resection (52.4%) was the most common surgery method, followed by multiple sublobar resection (34.8%). There was no difference in surgical strategy between patients with the same histological type and different histological types.

### 3.4. Molecular Status

Mutations in EGFR were tested in 291 lesions of 196 patients (58.3%) and the positive rate was 62.2%. Among the 181 EGFR positive lesions, L858 R missense mutation was the most common site (112, 61.9%), followed by exon 19 deletions (44, 24.3%). Concomitance of two or three EGFR mutations were present in 13 lesions. The detailed information of EGFR mutation is shown in Supplemental Table 2. In addition, the positive rates of ALK, ROS1, KRAS, and PD-L1 were 2.0%, 11.3%, 5.3%, and 11.6%, respectively (Table 3). Interestingly, patients with the same histology had a higher positive rate of EGFR (*p*=0.001) and ROS1 (*p*=0.01) and lower positive rate of PD-L1 (*p*=0.004).

### 3.5. Survival and Risk Factors of Overall Survival and Recurrence-Free Survival

**Figure 2:**
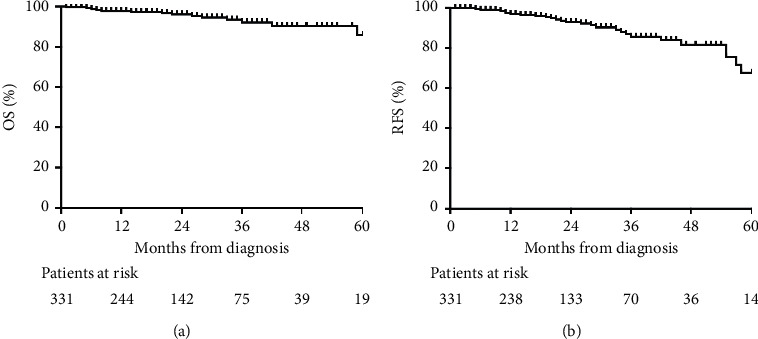
OS and RFS of overall patients. OS = overall survival; RFS = recurrence-free survival.

**Figure 3:**
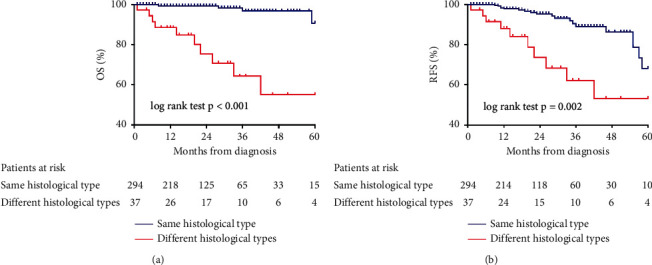
OS and RFS of patients with the same histological type and different histological types. OS = overall survival; RFS = recurrence-free survival.

Patients were followed up for a median period of 20 (11–34) months and 5 patients were lost to follow-up. During follow-up, 16 patients died, including 4 perioperative deaths and 12 deaths due to disease progression. The 1- and 3-year OS of overall patients were 97.7% and 92.2% (Figure 2(a)). The 1- and 3-year OS were 99.2% and 96.8% for patients with the same histological type, and 88.5% and 64.2% for patients with different histological types (*p* < 0.001) (Figure 3(a)). A total of 32 patients had recurrence. The 1- and 3-year RFS of overall patients were 96.8% and 85.7% (Figure 2(b)). The 1- and 3-year RFS were 97.8% and 89.0% for patients with the same histological type, and 88.1% and 62.1% for patients with different histological types (*p*=0.002) (Figure 3(b)).

Table 4 summarizes the univariable and multivariable analysis of clinicopathologic factors related to OS. In univariable analyses, ten variables were associated with OS. Four significant predictors were retained, following forward-stepwise variable selection, in the final multivariate Cox regression model. The factors associated with a worse OS were the presence of different histological types (adjusted hazard ratio (HR): 10.00; 95% CI: 2.92–34.48; *p* < 0.001), the older age at diagnosis (adjusted HR: 1.14; 95% CI: 1.04–1.26; *p*=0.008), pleural invasion (adjusted HR: 7.09; 95% CI: 1.42–35.71; *p*=0.017), and elevated neutrophile granulocyte (adjusted HR: 2.06; 95% CI: 1.30–3.27; *p*=0.002).

Table 5 summarizes the univariable and multivariable analysis of clinicopathologic factors associated with RFS. In univariable analyses, nine variables were associated with RFS. Four significant predictors were retained, following forward-stepwise variable selection, in the final multivariate Cox regression model. The factors associated with a worse RFS were the presence of different histological types (adjusted HR: 2.59; 95% CI: 1.14–5.88; *p*=0.023), the older age (adjusted HR: 1.06; 95% CI: 1.01–1.11; *p* = 0.023), pleural invasion (adjusted HR: 2.36; 95% CI: 1.07–5.18; *p*=0.032), and elevated neutrophile granulocyte (adjusted HR: 1.30; 95% CI: 1.01–1.69; *p*=0.032).

## 4. Discussion

In this study, we conducted an analysis of clinicopathologic characteristics and outcomes in 336 sMPLC patients. We demonstrated that in sMPLC, patients with different histological types only accounted for 11.6%. Importantly, although less common, patients with different histological types showed worse clinical characteristics and outcomes.

In patients with sMPLC, the histological types of different lesions are critical and some studies previously reported that patients with different histological types accounted for a proportion of 3.8%–61.5% [10–13]. However, the previous studies all had limited sample sizes. Our study with a relatively large cohort showed that patients with different histological types only accounted for 11.6% in sMPLC. It was speculated that the difference might be caused by the following reasons: different enrollment methods, regions, health awareness of residents, the popularity of physical examination, and surgical opportunities caused by the latest stage of sMPLC.

Remarkably, patients with different histological types showed an older age at diagnosis, more commonly males, more commonly with a smoking history and worse respiratory function than patients with the same histological type. These results hinted that the underlying mechanisms might be different between them. It was estimated that 75% of all lung cancers worldwide are smokers, consisting of 85% in men and 47% in women [16]. However, the rates of smokers in sMPLC seem to be lower and vary from 32.3% to 59.0% [12, 17, 18]. Furthermore, some studies reported that the rate of smokers in patients with multiple ground-glass nodules was much lower than that in patients with multiple solid nodules [18, 19]. All these findings suggested that smoking might have a lower impact on the development of sMPLC, especially for those with multiple ground-glass nodules. In our study, the rate of smokers was 26.2% in overall sMPLC, 16.8% in patients with the same histological type, and 79.5% in patients with different histological types. The relative low rate of smokers in our study was due to the major patients with the same histological type, which includes the majority of multiple ground-glass nodules. The different rates of smoking history between these two groups also suggest that smoking might not be the pathogenesis in patients with sMPLC with the same histology, but contribute to the development of sMPLC with different histological types.

The inflammatory process has been considered as an important mechanism of immune resistance, tumor growth and proliferation, and activation of cancer signaling pathway in cancer patients. Peripheral inflammatory state is related to poor prognosis of cancer patients [20]. A large number of routine blood parameters have been studied as potential biomarkers of inflammation in cancer patients, such as the increase of circulating leukocyte concentration, neutrophil absolute number, dNLR, and LDH level [21]. In this study, we demonstrated that dNLR and neutrophil absolute number were higher in patients with different histological types, and elevated neutrophil absolute value was significant predictor for worse OS and RFS, which indicated that inflammatory process might have a more important role in patients with different histological types.

Previous studies have revealed that large tumor size and lymph node involvement were independent factors for worse survival [22–24]. However, in our study, we initially reported that presence of different histological types was independent factors for worse OS and RFS. Large tumor size and advanced TNM stage were all significant in univariable analysis, but not significant in multivariable analysis. Instead, pleural invasion, another important parameter indicating disease progression, was independent factor for worse OS and RFS. For the overall patients in our study, the prognosis was relatively good after surgical resection compared to patients with metastatic and recurrent cancer [25, 26], which emphasized the importance for accurate diagnosis in clinical practice. However, patients with different histological types showed an obviously worse OS and RFS than patients with the same histological type and the presence of different histological types was significant predictor for worse OS and RFS. This might be explained by the following reasons. First, as discussed above, the underlying mechanisms between the two groups might be different, and inflammatory process and smoking had a more significant role in patients with different histological types. Second, patients with different histological types were diagnosed at an older age. Third, patients with different histological types showed worse clinical status at baseline with worse respiratory function and advanced stages. Due to the distinct OS and RFS between them, it is essential to distinguish them in clinical practice.

This study had some limitations. First, both Martini–Melamed and American College of Chest Physicians criteria are based on clinicopathological and radiological features of lung nodules. Despite their convenience to apply, they have notable limitations in the recognition of multiple intrapulmonary metastases. The forefront molecular approaches suggest their superiorities in distinguishing between MPLC and intro-pulmonary metastasis and there was a lack of in-depth molecular analysis of multiple lung lesions in this work. Second, this study was a single-center study. Third, due to the differences in clinical characteristics and genomics between Chinese and Western populations, our results need to be further studied and validated in Western patients. Last, the follow-up time span was not long enough and the numbers of events were relatively limited. Although the effect value was strong and statistically significant, considering that the wide confidence interval reflected the instability of the results, the interpretation of the results needed to be cautious and confirmed by further researches.

## 5. Conclusions

We demonstrated that in sMPLC, patients with different histological types only accounted for 11.6%. Importantly, although less common, patients with different histological types showed more severe clinical characteristics and lower recurrence-free survival and overall survival than patients with the same histological type.

## Figures and Tables

**Table 1:** Baseline clinical characteristics.

	Overall patients, *N* = 336	Same histological type, *N* = 297	Different histological types, *N* = 39	*p* value
*Demographics*				
Age, yrs	63 (56–70)	59 (52–65)	65 (62–69)	<0.001
Females	207 (61.6)	202 (68.0)	5 (12.8)	<0.001
BMI, kg/m^2^	22.9 (20.9–24.7)	22.8 (20.8–24.7)	23.8 (21.1–25.0)	0.315
*Symptoms*				
No symptom	232 (69.0)	215 (72.4)	17 (43.6)	<0.001
Respiratory symptom	72 (21.4)	58 (19.5)	14 (35.8)	0.019
Pain	21 (6.3)	17 (5.7)	4 (10.3)	0.285
Both	11 (3.3)	7 (2.4)	4 (10.3)	0.028
*Smoking history*	88 (26.2)	50 (16.8)	31 (79.5)	<0.001
*Lung function* (%)				
FEV1	102 (90–113)	104 (94–114)	89 (72–96)	<0.001
DLCO	100 (86–112)	102 (88–112)	85 (80–97)	<0.001
*Laboratory test*				
LDH	158 (139–176)	158 (139–176)	155 (143–180)	0.617
dNLR	1.47 (1.17–1.84)	1.44 (1.14–1.82)	1.73 (1.38–1.99)	0.013
Neutrophile granulocyte, 10^9^	3.16 (2.57–4.05)	3.11 (2.55–3.90)	4.02 (2.92–4.92)	0.002
*Features of lesions*				
Number				0.085
2	246 (73.2)	211 (71.0)	35 (89.7)	
3	57 (17.0)	53 (17.8)	4 (10.3)	
4	20 (6.0)	20 (6.8)	0 (0)	
>4	13 (3.9)	13 (4.4)	0 (0)	
Largest nodule size				
>2 cm	156 (46.4)	120 (40.4)	36 (92.3)	<0.001
>1 cm	294 (87.5)	255 (85.9)	39 (100)	0.008
Location				0.541
Ipsilateral	209 (62.2)	183 (61.6)	26 (66.7)	
Bilateral	127 (37.8)	114 (38.4)	13 (33.3)	

Values are *n* (%) or median (interquartile range). BMI = body mass index; DLCO = diffusing capacity for carbon monoxide; dNLR = derive neutrophil-to-lymphocyte radio; FEV1 = forced expiratory volume in 1 second; LDH = lactate dehydrogenase.

**Table 2:** Histological characteristics.

	Overall patients, *N* = 336	Same histological type, *N* = 297	Different histological types, *N* = 39	*p* value
*Pathologic characteristics*				
All adenocarcinoma	291 (86.6)	291 (98.0)	0 (0)	—
Adenocarcinoma + squamous	26 (7.7)	0 (0)	26 (66.7)	—
All squamous	6 (1.8)	6 (2.0)	0 (0)	—
Adenocarcinoma + others	11 (3.3)	0 (0)	11 (28.2)	—
Squamous + others	2 (0.6)	0 (0)	2 (5.1)	—
*TNM stage*				<0.001
I A1	63 (18.8)	63 (21.2)	0 (0)	
I A2	96 (28.6)	94 (31.6)	2 (5.1)	
I A3	46 (13.7)	42 (14.1)	4 (10.2)	
I B	83 (24.7)	78 (26.3)	5 (12.8)	
II A	12 (3.6)	9 (3.0)	3 (7.7)	
II B	17 (5.1)	9 (3.0)	8 (20.5)	
III A	13 (3.9)	2 (0.7)	11 (28.2)	
III B	4 (1.2)	0 (0)	4 (10.2)	
IV A	2 (0.6)	0 (0)	2 (5.1)	
*Pleural invasion*	108 (32.1)	86 (29.0)	22 (56.4)	0.001
Treatment				
Surgery timing				0.287
Concurrent	262 (78.0)	229 (77.1)	33 (84.6)	
Sequential	74 (22.0)	68 (22.9)	6 (15.4)	
Surgery interval, mon	7 [4–17]	7 [4–16]	9 [3–17]	0.564
Resect all nodules				0.263
Yes	122 (36.3)	111 (37.4)	11 (28.2)	
No	214 (63.7)	186 (62.6)	28 (71.8)	
Other treatments				
Chemotherapy	135 (40.2)	109 (36.7)	26 (66.6)	<0.001
Target therapy	11 (3.3)	11 (3.7)	0 (0)	0.624

Values are *n* (%) or median (interquartile range).

**Table 3:** Molecular status.

	All patients, *N* = 336	Same histological type, *N* = 297	Different histological types, *N* = 39	*p* value
EGFR				
Tested patients	196 (58.3)	180 (60.6)	16 (41.0)	—
Tested lesions	291	268	23	—
EGFR+	181 (62.2)	174 (64.9)	7 (30.4)	0.001
EGFR−	110 (37.8)	94 (35.1)	16 (69.6)	—
ALK				
Tested patient	290 (86.3)	257 (86.5)	33 (84.6)	—
Tested lesions	450	403	47	—
ALK+	9 (2.0)	8 (2.0)	1 (2.1)	0.947
ALK−	441 (98.0)	395 (98.0)	46 (97.9)	—
ROS1				
Tested patient	280 (83.3)	248 (83.5)	32 (82.1)	—
Tested lesions	433	387	46	—
ROS1+	49 (11.3)	49 (12.7)	0 (0)	0.01
ROS1−	231 (88.7)	338 (87.3)	46 (100)	—
KRAS				
Tested patient	35 (10.4)	33 (11.1)	2 (5.1)	—
Tested lesions	57	54	3	—
KRAS+	3 (5.3)	3 (5.6)	0 (0)	0.675
KRAS−	54 (94.7)	51 (94.4)	3 (100)	—
PD-L1				
Tested patient	190 (56.5)	172 (57.9)	18 (46.2)	—
Tested lesions	285	259	26	—
PD-L1+	33 (11.6)	25 (9.7)	8 (30.8)	0.004
PD-L1−	252 (88.4)	234 (90.3)	18 (69.2)	—

ALK = anaplastic lymphoma kinase; EGFR = epidermal growth factor receptor; KRAS = Kirsten-rat sarcoma 2 viral oncogene homolog; PD-L1 = programmed cell death ligand 1; ROS1 = ROS proto-oncogene 1.

**Table 4:** Univariable and multivariable analyses of clinicopathologic factors associated with OS.

Characteristics	Univariable analysis^*∗*^		Multivariable analysis^†^	
	HR (95% CI)	*p* value	HR (95% CI)	*p* value
Age, yrs	1.10 (1.03–1.18)	0.005	1.14 (1.04–1.26)	0.008
Females	10.07 (2.28–44.49)	0.002		
Histology, different vs. same	12.29 (4.44–34.03)	<0.001	10.00 (2.92–34.48)	<0.001
Smoking history	19.12 (4.33–84.34)	<0.001		
FEV1	0.98 (0.95–0.99)	0.033		
DLCO	0.99 (0.96–1.01)	0.333		
dNLR	1.82 (1.10–3.02)	0.021		
Neutrophile granulocyte, 10^9^	1.97 (1.42–2.73)	<0.001	2.06 (1.30–3.27)	0.002
Number of lesions, >2 vs. 2	1.01 (0.32–3.18)	0.987		
Largest nodule size, >2 vs. <2 cm	5.05 (1.43–17.81)	0.012		
TNM stage, IB-IV vs. IA	7.87 (1.77–34.48)	0.007		
Pleural invasion	9.43 (2.10–41.67)	0.003	7.09 (1.42–35.71)	0.017

BMI = body mass index; DLCO = diffusing capacity for carbon monoxide; dNLR = derive neutrophil-to-lymphocyte radio; FEV1 = forced expiratory volume in 1 second; LDH = lactate dehydrogenase; OS = overall survival.

**Table 5:** Univariable and multivariable analyses of clinicopathologic factors associated with RFS.

Characteristics	Univariable analysis^*∗*^		Multivariable analysis^†^	
	HR (95% CI)	*p* value	HR (95% CI)	*p* value
Age, yrs	1.06 (1.02–1.11)	0.010	1.06 (1.01–1.11)	0.023
Females	2.10 (1.02–4.34)	0.045		
Histology, different vs. same	2.96 (1.39–6.33)	0.005	2.59 (1.14–5.88)	0.023
Smoking history	2.29 (1.14–4.63)	0.020		
FEV1	1.00 (0.99–1.02)	0.855		
DLCO	1.01 (0.99–1.03)	0.314		
dNLR	1.59 (1.05–2.40)	0.028		
Neutrophile granulocyte, 10^9^	1.47 (1.14–1.89)	0.003	1.30 (1.01–1.69)	0.046
Number of lesions, >2 vs. 2	1.69 (0.82–3.47)	0.156		
Largest nodule size, >2 vs. <2 cm	2.27 (1.09–4.72)	0.028		
TNM stage, IB-IV vs. IA	2.60 (1.19–5.68)	0.016		
Pleural invasion	2.81 (1.31–6.02)	0.008	2.36 (1.07–5.18)	0.032

BMI = body mass index; DLCO = diffusing capacity for carbon monoxide; dNLR = derive neutrophil-to-lymphocyte radio; FEV1 = forced expiratory volume in 1 second; LDH = lactate dehydrogenase; RFS = recurrence-free survival.

## Data Availability

The data used to support the findings of this study are available from the corresponding author upon request.

## Supplementary Materials

Supplementary Materials
*Supplementary Table 1.* Surgical strategy. *Supplemental Table 2.* Detailed information of EGFR mutation.
