# Supplementary material for: Clinicopathologic Characteristics and Outcomes of Simultaneous Multiple Primary Lung Cancer
Source: J Oncol. 2021 Dec 23;2021:7722231. doi: 10.1155/2021/7722231 (PMC8718277; doi:10.1155/2021/7722231)
Supplement: Supplementary Materials — Supplementary Table 1. Surgical strategy. Supplemental Table 2. Detailed information of EGFR mutation. [file 7722231.f1.docx]

**Supplementary Appendix**

**Clinicopathologic characteristics and outcomes of simultaneous multiple primary lung cancer**

Ying Liu, Yu-Ping Zhou, Mai Zhang, Li Li, Hu Liao, Lin Ma, Feng Lin, Yue-Yun Chen, Chen-Xi Fu, Ting-Ting Huang, You Lu, Yan Zhang

| **Supplemental Table 1. Surgical strategy** | | | | |
| --- | --- | --- | --- | --- |
|  | Overall patients  N = 336 | Same Histological type  N = 297 | Different Histological types  N = 39 | P Value |
| Surgery timing |  |  |  | 0.287 |
| Concurrent | 262 (78.0) | 229 (77.1) | 33 (84.6) |  |
| Sequential | 74 (22.0) | 68 (22.9) | 6 (15.4) |  |
| Surgical path |  |  |  | 0.131 |
| Open heart surgery | 71 (21.1) | 67 (22.6) | 4 (10.3) |  |
| Thoracoscopic surgery | 247 (73.5) | 213 (71.7) | 34 (87.2) |  |
| Both | 18 (5.4) | 17 (5.7) | 1 (2.5) |  |
| Surgery methods |  |  |  | 0.609 |
| Single lobectomy | 17 (5.0) | 14 (4.7) | 3 (7.7) |  |
| Multiple Lobectomy | 20 (6.0) | 16 (5.4) | 4 (10.3) |  |
| Single Sublobar resection | 4 (1.2) | 4 (1.3) | 0 (0) |  |
| Multiple Sublobar resection | 117 (34.8) | 106 (35.7) | 11 (28.2) |  |
| Lobectomy + Sublobar resection | 176 (52.4) | 155 (52.2) | 21 (53.8) |  |
| Total pneumonectomy | 2 (0.6) | 2 (0.7) | 0 (0) |  |
| Values are n (%) or median (interquartile range). | | | | |

| **Supplemental Table 2. Detailed information of EGFR mutation.** | |
| --- | --- |
| EGFR mutation | Number of lesions |
| L858R | 112 |
| 19DEL | 44 |
| 20INS | 4 |
| L861Q | 3 |
| G719X | 3 |
| G719A | 1 |
| G719S | 1 |
| 19DEL-T790M | 2 |
| L858R-T790M | 2 |
| 19DEL-L858R | 1 |
| 19DEL-L861Q | 1 |
| L858R-G719X | 1 |
| L858R-S768I | 1 |
| L858R-A871G | 1 |
| G719X-S768I | 1 |
| G719X-L861Q | 1 |
| L833V-H835L | 1 |
| L858R-G719X-S768I | 1 |
| EGFR = epidermal growth factor receptor | |
